# Functional Convergence Spasm and Dysconjugate Eye Movements: A Vignette

**DOI:** 10.1002/mdc3.70318

**Published:** 2025-08-26

**Authors:** Iryna Klopotovska, Koemi‐Jade Garrick, Diego Kaski

**Affiliations:** ^1^ University College London Hospitals NHS Foundation Trust London United Kingdom; ^2^ Royal National Ear Nose and Throat Hospital, UCLH London United Kingdom; ^3^ UCL Queen Square Neurology London United Kingdom

**Keywords:** functional, eye movement disorder, convergence spasm, dysconjugacy, saccade

A 46‐year‐old woman presented with a 3‐month history of intermittent diplopia, blurred vision, and unsteadiness, following a sudden episode of disorientation and collapse while supervising an examination. After this collapse, she developed increased sound sensitivity and worsening motion sickness. She had no prior medical history or regular medications. Her family history included multiple sclerosis and brain tumor. Examination was limited due to visual discomfort. Eye movements were full, with no spontaneous or gaze‐evoked nystagmus, but were often dysconjugate, showing brief paroxysms of convergent effort—especially during saccades and pursuit—accompanied by pupillary constriction (see Video 1). Vestibulo‐ocular reflex (VOR) was intact. Dix‐Hallpike and roll test elicited no nystagmus, though convergence effort occurred intermittently. Gait was broad‐based and cautious, with prolonged single‐leg stance time. She took multiple steps on the pull test and had a positive shoulder tap sign. Based on the above, a diagnosis of functional gait disorder and functional convergence spasm with dysconjugate eye movements was made. Magnetic resonance imaging (MRI) was normal. The patient was educated on the diagnosis and referred to physiotherapy and ocular motor rehabilitation.

**VIDEO 1:** Right gaze—dysconjugacy to the right with impaired abduction of the right eye and convergent effort in the left eye. Central gaze—microsaccadic oscillations (square wave jerks). Left gaze—dysconjugacy to the left with impaired abduction of the left eye and convergent effort in the right eye. Up gaze, down gaze—convergence spasm in both eyes on attempted up and downward gaze. Note also pupillary miosis (part of the accommodation reflex). *Horizontal saccadic stimuli—*saccadic dysconjugacy with impaired and irregular abduction to the right and left. Smooth pursuit—dysconjugate eye movements during attempted target tracking.

Conjugate horizontal saccades rely on a brainstem circuit involving burst neurons in the paramedian pontine reticular formation (PPRF), normally inhibited by omnipause neurons in the nucleus raphe interpositus. When omnipause neurons stop firing, burst neurons activate the abducens nucleus, stimulating the ipsilateral lateral rectus and contralateral medial rectus via internuclear neurons through the medial longitudinal fasciculus (MLF), enabling conjugate movement.^1^ Despite near‐perfect coordination, slight physiological dysconjugacies can occur. Early differences (within 5 ms) may reflect internuclear transmission delay, whereas later‐phase disparities often relate to biomechanical factors like greater peak velocity in the abducting eye.^1^

Functional convergence spasm (CS) is a functional eye movement disorder characterized by involuntary convergence, accommodation, and miosis without a near stimulus. It is the most frequently reported functional ocular motor abnormality.^2^ Pupillary constriction, as shown in our patient, is a key feature suggesting accommodative effort rather than a deficit.^3^ CS may be triggered during sustained gaze, as demonstrated in Video 1. Other neurological causes of convergence spasm are rare and include lesions in the diencephalic–mesencephalic junction (eg, thalamic esotropia), Wernicke‐Korsakoff syndrome, and posterior fossa pathology.^4^

In our patient, videonystagmography (VNG) revealed dysconjugate movements unrelated to visual stimuli, often mistaken for abducens palsy or internuclear ophthalmoplegia (INO). Our patient demonstrated variability in excessive abduction (convergence spasm) affecting either eye, consistent with the variability reported in functional movement disorders (Fig. 1). Management focuses on patient education, reassurance, and structured rehabilitation (that may include eye movement retraining), with cognitive behavioral therapy or psychological support when necessary. The use of mydriatics has been suggested for convergence spasm, but there is very limited evidence to support their use,^5^ and anecdotally, we have not had success with these.

**FIG. 1:**
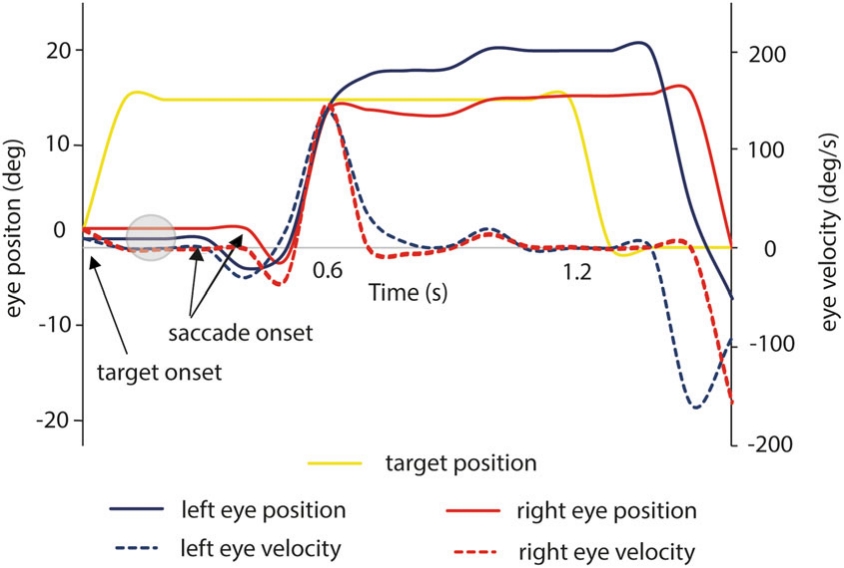
A representative time plot of a single 15‐degree horizontal saccade to the right from a patient with functional dysconjugate eye movements generated from the videonystagmography (VNG) recording. The plot shows eye position (continuous lines) and eye velocities (dashed lines). Note increased saccadic latency (saccade onset [red and blue traces] relative to target motion [yellow trace]), which is a recognized feature functional eye movement disorder.^5^ Normal saccadic latency is circa. 150–200 ms (gray circle). The initiation of the saccade is almost simultaneous in both eyes (red and blue traces leave the horizontal axis around the same time point, circa 0.4 s). However, dysconjugation appears later due to excessive adduction of the left eye (separation of blue and red eye position traces from 0.6 s), consistent with convergence spasm in the left eye. Initial negative values (around 0.3 s) are due to small vergence eye movements prior to saccade onset. Negative values represent leftward directed eye movements.

## Author Roles

(1) Research project: A. Conception, B. Organization, C. Execution; (2) Statistical analysis: A. Design, B. Execution, C. Review and critique; (3) Manuscript: A. Writing of the first draft, B. Review and critique.

I.K.: 1B, 1C, 3A

K.J.G.: 1B, 1C

D.K.: 1A, 1B, 1C, 1B, 3B

## Disclosures

**Ethical Compliance Statement:** The authors confirm that approval of an institutional review board was not required for this work. The patient provided written consent for eye recordings using videonystamography. We confirm that we have read the journal's position on issues involved in ethical publication and affirm that this work is consistent with those guidelines.

**Funding Sources and Conflicts of Interest**: No specific funding was received for this work. The authors declare that there are no conflicts of interest relevant to this work.

**Financial Disclosures for the Previous 12 Months:** I.K. is supported by a Clinical Fellowship grant from the European Academy of Neurology. D.K. is supported by the National Institute for Health Research University College London Hospitals Biomedical Research Centre.

## Data Availability

The data that supports the findings of this study are available in the supplementary material of this article.
